# Therapeutic efficacy of artesunate–amodiaquine and artemether–lumefantrine combinations for uncomplicated malaria in 10 sentinel sites across Ghana: 2015–2017

**DOI:** 10.1186/s12936-019-2848-1

**Published:** 2019-06-24

**Authors:** Benjamin Abuaku, Nancy O. Duah-Quashie, Lydia Quaye, Sena A. Matrevi, Neils Quashie, Akosua Gyasi, Felicia Owusu-Antwi, Keziah Malm, Kwadwo Koram

**Affiliations:** 1 Epidemiology Department, Noguchi Memorial Institute for Medical Research, College of Health Sciences, University of Ghana, P. O. Box LG581, Legon, Accra, Ghana; 2 Centre for Tropical Clinical Pharmacology and Therapeutics, University of Ghana Medical School, P. O. Box GP4236, Accra, Ghana; 3 National Malaria Control Programme, Public Health Division, Ghana Health Service, Accra, Ghana; 4 World Health Organization, Country Office, Accra, Ghana

**Keywords:** Therapeutic efficacy, Artesunate–amodiaquine, Artemether–lumefantrine, Uncomplicated malaria, Sentinel sites, Ghana

## Abstract

**Background:**

Routine surveillance on the therapeutic efficacy of artemisinin-based combination therapy (ACT) has been ongoing in Ghana since 2005. The sixth round of surveillance was conducted between 2015 and 2017 to determine the therapeutic efficacy of artesunate–amodiaquine (AS–AQ) and artemether–lumefantrine (AL) in 10 sentinel sites across the country.

**Methods:**

The study was a one-arm, prospective, evaluation of the clinical, parasitological, and haematological responses to directly observed treatment with AS–AQ and AL among children 6 months to 9 years old with uncomplicated falciparum malaria. The WHO 2009 protocol on surveillance of anti-malaria drug efficacy was used for the study with primary outcomes as prevalence of day 3 parasitaemia and clinical and parasitological cure rates on day 28. Secondary outcomes assessed included patterns of fever and parasite clearance as well as changes in haemoglobin concentration.

**Results:**

Day 3 parasitaemia was absent in all sites following treatment with AS–AQ whilst only one person (0.2%) was parasitaemic on day 3 following treatment with AL. Day 28 PCR-corrected cure rates following treatment with AS–AQ ranged between 96.7% (95% CI 88.5–99.6) and 100%, yielding a national rate of 99.2% (95% CI 97.7–99.7). Day 28 PCR-corrected cure rates following treatment with AL ranged between 91.3% (95% CI 79.2–97.6) and 100%, yielding a national rate of 96% (95% CI 93.5–97.6). Prevalence of fever declined by 88.4 and 80.4% after first day of treatment with AS–AQ and AL, respectively, whilst prevalence of parasitaemia on day 2 was 2.1% for AS–AQ and 1.5% for AL. Gametocytaemia was maintained at low levels (< 5%) during the 3 days of treatment. Post-treatment mean haemoglobin concentration was significantly higher than pre-treatment concentration following treatment with either AS–AQ or AL.

**Conclusions:**

The therapeutic efficacy of AS–AQ and AL is over 90% in sentinel sites across Ghana. The two anti-malarial drugs therefore remain efficacious in the treatment of uncomplicated malaria in the country and continue to achieve rapid fever and parasite clearance as well as low gametocyte carriage rates and improved post-treatment mean haemoglobin concentration.

**Electronic supplementary material:**

The online version of this article (10.1186/s12936-019-2848-1) contains supplementary material, which is available to authorized users.

## Background

Malaria control efforts in Ghana have, over the years, yielded some positive results. The national parasite prevalence among children aged 6–59 months decreased from 27.5% in 2011 to 20.4% in 2016 [1, 2]. The proportion of out-patients suspected to have malaria decreased from 44% in 2013 to 34% in 2017 [3, 4]. Malaria parasite positivity rates among febrile cases in 30 sentinel sites have also shown a significant decline from 23.7% in 2014 to 16.7% in 2017 (unpublished data). The proportion of deaths that were attributable to malaria declined from 19.5% in 2010 to 2.1% in 2017 [4, 5]. Despite the gains made in control efforts, malaria remains a major public health problem in Ghana accounting for an annual economic loss of approximately US$6.6 million among businesses in the country [6].

Currently, case management remains one of the main malaria interventions in Ghana. There are three first-line anti-malarial drugs for treating uncomplicated malaria in the country. These are artesunate–amodiaquine (AS–AQ) combination, artemether–lumefantrine (AL) combination, and dihydroartemisinin–piperaquine (DHAP) combination [7]. AL or DHAP is the drug of choice for confirmed uncomplicated malaria following seasonal malaria chemoprevention (SMC), which has been piloted and scaled up in the northern savannah zone of the country using amodiaquine–sulfadoxine/pyrimethamine (AQ–SP) combination [7, 8]. The emergence and spread of both artemisinin and partner drug resistance threatens the efficacy of artemisinin-based combination therapy (ACT) and subsequently undermines the clinical objective of treating uncomplicated malaria, which is to eliminate all parasites from the body and prevent progression to severe disease [9, 10]. It is, therefore, necessary to provide continuous data on the therapeutic efficacy of first-line ACT to ensure real-time evidence-based review of national treatment policies as and when necessary. Since the introduction of ACT in Ghana in 2005, there have been five rounds of national surveillance on therapeutic efficacy [11–14]. This paper presents data on the therapeutic efficacy of AS–AQ and AL combinations from the sixth round of surveillance conducted between August 2015 and December 2017 in 10 sentinel sites across the country using the 2009 WHO protocol for surveillance of anti-malarial drug efficacy [15]. Efficacy of DHAP was not studied during this round of surveillance because it was cost-effective focusing on the widely supplied and used ACT (AS–AQ and AL) in the country [4].

## Methods

### Study design

The study was a one-arm, prospective, evaluation of the clinical, parasitological and haematological responses to directly observed therapy for uncomplicated malaria among children aged 6 months to 9 years in 10 sentinel sites across Ghana. Primary objectives were to assess prevalence of day 3 parasitaemia as well as the clinical and parasitological cure rates on day 28 following treatment with AS–AQ and AL. Secondary objectives were to assess the patterns of fever and parasite clearance, changes in haemoglobin concentration, gametocyte carriage rates, and prevalence of adverse events. Five of the 10 sentinel sites were scheduled to first study AS–AQ followed by AL whilst the other five were scheduled to first study AL followed by AS–AQ.

### Study sites

**Fig. 1:**
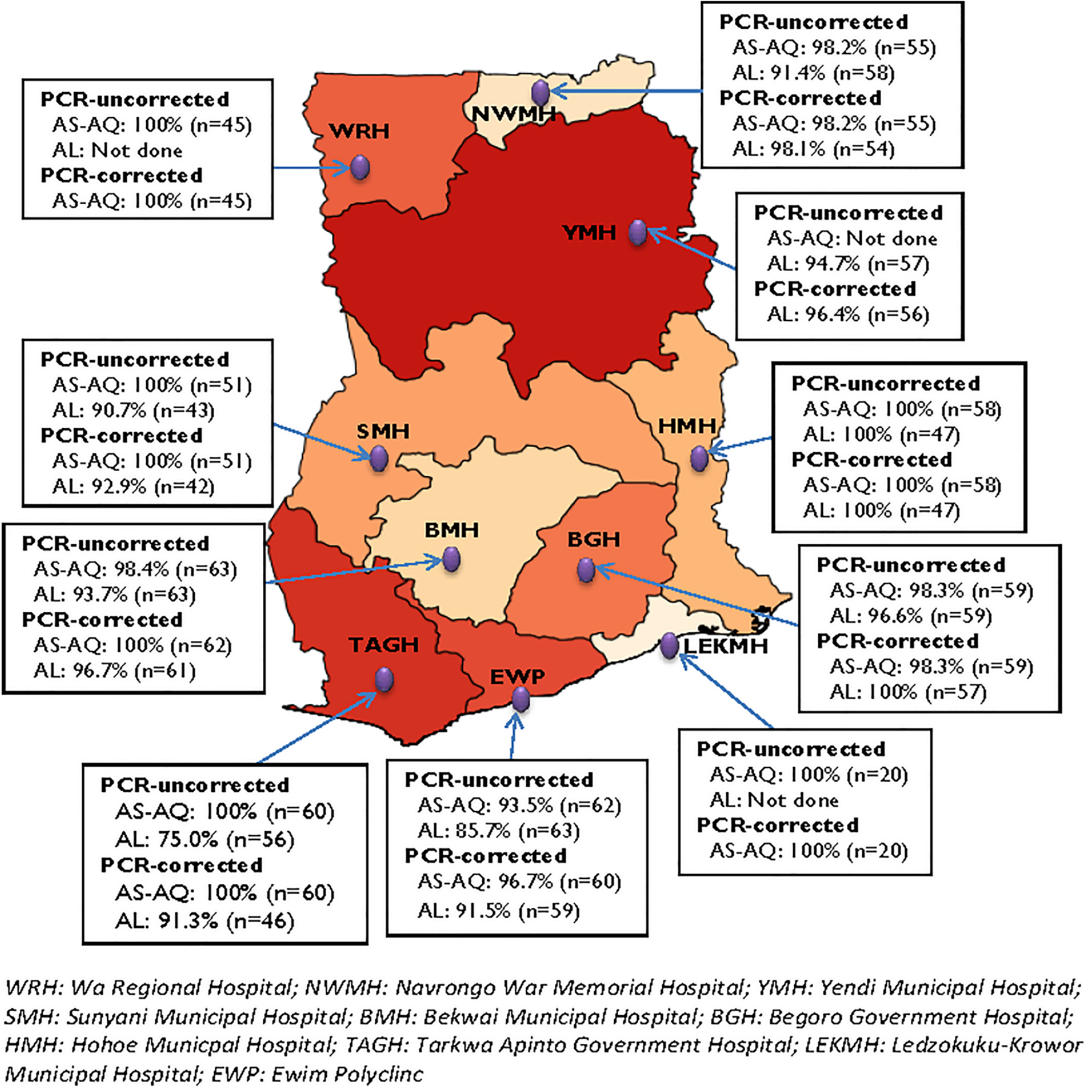
Map of Ghana showing sentinel sites for monitoring antimalarial drug efficacy and corresponding PCR-uncorrected and PCR-corrected cure rates for AS–AQ and AL

The study was conducted in all 10 sentinel sites for anti-malarial drug efficacy monitoring in Ghana (Fig. 1). The sites were Navrongo War Memorial Hospital (NWMH), Yendi Municipal Hospital (YMH) and Wa regional Hospital (WRH), located within the northern savannah zone of the country; Sunyani Municipal Hospital (SMH), Bekwai Municipal Hospital (BMH), Begoro Government Hospital (BGH), Hohoe Municipal Hospital (HMH), and Tarkwa Apinto Government Hospital (TAGH), located within the forest zone of the country; and, Ledzokuku Krowor Municipal Hospital (LEKMH) and Ewim Polyclinic (EWP) located, within the coastal zone of the country. Malaria transmission in the northern savannah zone is perennial with marked seasonal variation and estimated annual entomological inoculation rate (EIR) of up to 1132 infective bites per person per year [16–18]. Malaria transmission in the forest zone is intense and perennial with estimated annual EIR of up to 866 infective bites per person per year [17, 19, 20]. Malaria transmission in the coastal zone is perennial but not intense with estimated annual EIR of fewer than 50 infective bites per person per year [21].

Seven sites (NWMH, SMH, HMH, BMH, BGH, TAGH, EWP) were able to study both AS–AQ and AL during the study period whilst WRH and LEKMH studied only AS–AQ, and YMH studied only AL.

### Study population

The study population was made up of febrile children aged 6 months to 9 years suspected to have malaria. Inclusion criteria were axillary temperature ≥ 37.5 °C or history of fever during the past 24 h, mono-infection with *Plasmodium falciparum,* parasite count ranging between 1000 and 250,000/µl, haemoglobin level > 5 g/dl, parental consent, ability and willingness to comply with study protocol, absence of signs/symptoms of severe malaria, absence of severe malnutrition, absence of other causes of fever such as pneumonia, otitis media, gastroenteritis, measles, and urinary tract infection, absence of chronic disease such as cardiac, renal and hepatic, and, history of allergy to test medicine.

### Treatment

All anti-malarials used in the study were fixed-dose combinations, and were supplied by WHO, Geneva. The AS–AQ used were from Sanofi Aventis whilst the AL used were from Ipca Laboratories Ltd, India. Treatment doses for AS–AQ were as follows: children weighing: (i) 4.5 to < 9 kg received a tablet of the 25/67.5 mg product daily for 3 days; (ii) 9 to < 18 kg received a tablet of the 50/135 mg product daily for 3 days; and, (iii) 18 to < 36 kg received a tablet of the 100/270 mg product daily for 3 days. The administration of AL (20/120 mg), which was also by weight, was at 0, 8, 24, 36, 48, and 60 h. Treatment doses were as follows: children weighing: (i) 5 to < 15 kg received one tablet per treatment hour; (ii) 15 to < 25 kg received two tablets per treatment hour; and, (iii) 25 to < 35 kg received three tablets per treatment hour. All treatments were given under direct observation of a study nurse after satisfying himself/herself that the child had eaten. The child was then observed for 30 min to ascertain retention of anti-malarial. Children who vomited during the observation period were re-treated with the same dose of anti-malarial and observed for an additional 30 min. Children with repeated vomiting were withdrawn from the study and treated as severe malaria according to national standard treatment guidelines using the following options: (i) intravenous artesunate (2.4 mg and 3.0 mg/kg body weight for children weighing ≥ 20 kg and < 20 kg, respectively, on admission, then at 12, 24 h, and daily until patient was able to swallow); or, (ii) intramuscular artemether (3.2 mg/kg body weight as loading dose and 1.6 mg/kg body weight once daily until patient was able to swallow). Parenteral treatment of severe malaria cases was given for a minimum of 24 h. A full 3-day course of oral ACT was given when child was able to swallow oral medication [7]. All children in the study were allowed use of antipyretics (acetaminophen).

### Patient follow-up

All children enrolled were scheduled to visit the outpatient clinic on days 1, 2, 3, 7, 14, 21, and 28 (day 0 being the day treatment was started). Enrolled children were clinically examined by the study physician any time they visited the clinic. Asexual parasite densities and presence of gametocytes were assessed on days 2, 3, 7, 14, 21, 28, and any unscheduled visit within the 28 days of follow-up.

### Laboratory procedures

Thick and thin blood smears prepared on the days of parasitological assessment were stained with 3% Giemsa for 30–45 min. Asexual parasites were counted against 200 white blood cells using a hand tally counter whilst the presence of gametocytes was noted. Parasite densities were finally expressed per µl blood assuming white blood cell count of 8000/µl blood. A blood smear was declared negative after examination of 100 thick-film fields. All blood slides were read by two qualified independent microscopists, and discordant readings (in terms of presence or absence of parasites: asexual or sexual, species identification, and day 0 count meeting the inclusion criterion of 1000–250,000/µl blood) were re-examined by a third qualified independent microscopist, whose reading was considered final. Filter paper blots were obtained on day 0 and at recurrence of parasitaemia for PCR genotyping using merozoite surface protein 2 (MSP2)-specific primers: FC 27 and 3D7 to distinguish between recrudescence and re-infection [22, 23]. Samples were classified as recrudescence when pre-treatment and post-treatment alleles had the same band sizes (base pairs). Haemoglobin levels were assessed for all study children on days 0 and 28 using an automated haematology analyzer (Sysmex KX-21N™).

### Data analysis

A minimum sample size of 50 was computed for each sentinel site based on an estimated treatment failure rate of 5% at 95% confidence level, 10% precision, and 20% loss to follow-up. Data were captured using WHO Microsoft Excel^®^ template for therapeutic efficacy tests [15]. Primary treatment outcomes analysed for each ACT were prevalence of day 3 parasitaemia and day 28 PCR-uncorrected and PCR-corrected outcomes as per WHO criteria: early treatment failure (ETF), late parasitological failure (LPF), late clinical failure (LCF), and adequate clinical and parasitological response (ACPR) [15]. Day 28 treatment outcomes were analysed per protocol and Kaplan–Meier. Briefly, PCR-uncorrected per protocol analysis excluded children lost to follow-up and withdrawn whilst Kaplan–Meier analysis censored last day of follow-up for such children. PCR-corrected per protocol analysis excluded children lost to follow-up, withdrawn, with falciparum re-infection, and undetermined PCR whilst Kaplan–Meier analysis censored last day of follow-up for those lost to follow-up as well as those withdrawn or with falciparum re-infection. Children with undetermined PCR were also excluded in the Kaplan–Meier analysis [15]. Secondary treatment outcomes analysed were patterns of fever (i.e. temperature ≥ 37.5 °C) and parasite clearance during the first week of follow-up, mean differences between pre- (day 0) and post-treatment (day 28) haemoglobin concentration, and prevalence of adverse events. Chi-square and Fisher’s exact tests were used to compare proportions whilst Student’s t-test was used to compare means (significant at p < 0.05).

## Results

### Baseline characteristics

Out of a total of 1463 children screened in nine facilities to receive AS–AQ, 492 met the inclusion criteria and were enrolled (Additional file 1). Apart from one facility (LEKMA), male/female ratio for those treated with AS–AQ was approximately 1:1 with majority of the children (283/492 or 57.5%) under 5 years old. Mean weight ranged between 14.4 kg (± 5.3) in WRH and 17.9 kg (± 6.0) in NWMH, yielding an overall mean of 16.1 kg (± 5.5). Mean axillary temperature ranged between 37.7 °C (± 0.9) in SMH and 38.7 °C (± 0.8) in NWMH, yielding an overall mean of 38.1 °C (± 1.1). Geometric mean parasitaemia ranged between 9039/µl blood in TAGH and 53,5441/µl blood in NMWH, yielding an overall mean of 31,119/µl blood. Gametocytaemia was prevalent in four facilities. Mean haemoglobin concentration ranged between 8.8 g/dl (± 1.8) in WRH and 10.8 g/dl (± 1.9) in BGH, yielding an overall mean of 10.1 g/dl (± 1.8) (Table 1).

**Table 1:** Demographic, clinical, parasitological, and haematological characteristics of patients treated with AS–AQ at baseline

Characteristic	NWMH	WRH	SMH	BMH	BGH	HMH	TAGH	LEKMH	EWP	TOTAL
	n = 55	n = 51	n = 54	n = 63	n = 62	n = 61	n = 61	n = 23	n = 62	N = 492
Male/female	24/31	27/24	28/26	30/33	25/37	33/28	38/23	7/16	32/30	244/248
Age group										
< 5 years (%)	21 (38.2)	37 (72.5)	35 (64.8)	44 (69.8)	32 (51.6)	37 (60.7)	34 (55.7)	14 (60.9)	29 (46.8)	283 (57.5)
5–9 years	34	14	19	19	30	24	27	9	33	209
Weight (kg)										
Mean weight (sd)	17.9 (6.0)	14.4 (5.3)	16.1 (5.1)	14.7 (4.9)	16.0 (6.1)	15.5 (4.6)	16.0 (5.4)	17.6 (5.5)	17.2 (6.0)	16.1 (5.5)
Range (min, max)	8.0, 30.0	7.1, 27.0	7.0, 27.0	6.6, 35.0	6.1, 32.3	8.0, 27.0	7.2, 29.0	9.0, 29.4	6.7, 30.0	6.1, 35.0
Axillary temperature in °C										
Mean temperature (sd)	38.7 (0.8)	37.9 (1.0)	37.7 (0.9)	38.2 (1.1)	37.7 (1.1)	38.0 (1.0)	38.6 (1.1)	38.1 (1.2)	38.2 (1.1)	38.1 (1.1)
Range (min, max)	37.5, 40.4	36.0, 39.8	36.0, 39.8	35.4, 40.3	35.2, 39.7	36.2, 40.1	36.0, 40.4	35.9, 39.7	35.8, 40.5	35.2, 40.5
Asexual parasitaemia/µl										
Geometric mean	53,441	42,754	31,815	39,961	29,423	36,917	9039	18,747	41,089	31,119
Range (min, max)	3200, 217,182	1200, 247,040	1391, 208,000	2365, 246,227	1822, 146,686	1747, 206,502	1000, 163,343	3248, 101,423	1340, 229,410	1000, 247,040
Gametocytaemia (%)	0.0	2.0	0.0	0.0	0.0	1.6	1.6	0.0	3.2	1.0
Haemoglobin level in g/dl										
Mean (sd)	9.6 (1.6)	8.8 (1.8)	10.0 (1.7)	10.3 (2.0)	10.8 (1.9)	10.1 (1.4)	10.5 (1.6)	9.9 (1.7)	10.8 (1.4)	10.1 (1.8)
Range (min, max)	6.0, 12.7	5.4, 12.8	6.0, 12.9	6.4, 16.0	5.1, 13.9	6.5, 13.3	6.1, 13.1	7.1, 12.2	7.2, 13.9	5.1, 16.0

*sd* standard deviation, *NWMH* Navrongo War Memorial Hospital, *WRH* Wa Regional Hospital, *SMH* Sunyani Municipal Hospital, *BMH* Bekwai Municipal Hospital, *BGH* Begoro Government Hospital, *HMH* Hohoe Municipal Hospital, *TAGH* Tarkwa Apinto Government Hospital, *LEKMH* Ledzokuku-Krowor Municipal Hospital, *EWP* Ewim Polyclinic

Out of a total of 1068 children screened in eight facilities to receive AL, 472 met the inclusion criteria and were enrolled. Male/female ratio for those treated with AL was approximately 1:1 in all sites with majority of the children (267/472 or 56.6%) under 5 years old. Mean weight ranged between 14.0 kg (± 6.0) in YMH and 17.8 kg (± 5.3) in HMH, yielding an overall mean of 16.0 kg (± 5.7). Mean axillary temperature ranged between 37.1 °C (± 0.8) in SMH and 38.5 °C (± 0.8) in NWMH, yielding an overall mean of 38.0 °C (± 1.1). Geometric mean parasitaemia ranged between 2941/µl blood in TAGH and 54,373/µl blood in BGH, yielding an overall mean of 23,534/µl blood. Gametocytaemia was prevalent in only one facility (BMH). Mean haemoglobin concentration ranged between 9.0 g/dl (± 1.5) in YMH and 10.9 g/dl (± 1.8) in TAGH, yielding an overall mean of 10.1 g/dl (± 1.6) (Table 2).

**Table 2:** Demographic, clinical, parasitological, and haematological characteristics of patients treated with AL at baseline

Characteristic	NWMH	YMH	SMH	BMH	BGH	HMH	TAGH	EWP	TOTAL
	n = 58	n = 60	n = 43	n = 63	n = 63	n = 60	n = 58	n = 67	N = 472
Male/female	29/29	32/28	20/23	34/29	32/31	33/27	32/26	37/30	249/223
Age group									
< 5 years (%)	33 (56.9)	33 (55.0)	32 (74.4)	45 (71.4)	34 (54.0)	19 (31.7)	37 (63.8)	34 (50.7)	267 (56.6)
5–9 years	25	27	11	18	29	41	21	33	205
Weight (kg)									
Mean weight (sd)	15.9 (5.2)	14.0 (6.0)	15.4 (4.7)	14.1 (4.3)	17.3 (6.9)	17.8 (5.3)	16.2 (5.9)	17.1 (5.8)	16.0 (5.7)
Range (min, max)	8.0, 29.0	5.0, 35.0	8.0, 30.0	8.0, 26.0	7.0, 35.0	7.8, 32.0	6.2, 33.0	7.0, 40.0	5.0, 40.0
Axillary temperature in °C									
Mean temperature (sd)	38.5 (1.2)	37.6 (0.9)	37.1 (0.8)	38.3 (1.2)	38.0 (1.3)	38.2 (0.9)	38.3 (1.0)	38.0 (1.1)	38.0 (1.1)
Range (min, max)	36.1, 40.5	36.0, 39.5	35.5, 39.8	35.7, 41.5	35.8, 40.8	37.0, 40.8	35.7, 40.2	36.1, 40.7	35.5, 41.5
Asexual parasitaemia/µl									
Geometric mean	14,454	36,433	23,957	21,975	54,373	40,198	2941	44,238	23,534
Range (min, max)	2200, 183,680	1035, 218,877	1040, 211,400	2528, 215,615	5963, 226,615	5040, 24,783	1120, 65,800	1200, 249,960	1035, 249,960
Gametocytaemia (%)	0.0	0.0	0.0	1.6	0.0	0.0	0.0	0.0	0.2
Haemoglobin level in g/dl									
Mean (sd)	9.5 (1.6)	9.0 (1.5)	10.4 (1.2)	9.7 (1.3)	10.4 (1.4)	10.2 (1.1)	10.9 (1.8)	10.8 (1.7)	10.1 (1.6)
Range (min, max)	6.2, 11.9	5.4, 13.4	7.3, 12.5	6.4, 13.0	7.2, 13.8	7.1, 12.3	7.7, 15.2	6.5, 16.6	5.4, 16.6

*sd* standard deviation, *NWMH* Navrongo War Memorial Hospital, *YMH* Yendi Municipal Hospital, *SMH* Sunyani Municipal Hospital, *BMH* Bekwai Municipal Hospital, *BGH* Begoro Government Hospital, *HMH* Hohoe Municipal Hospital, *TAGH* Tarkwa Apinto Government Hospital, *EWP* Ewim Polyclinic

### Primary outcomes

For all sites that tested AS–AQ, there was no child with parasitaemia on day 3. For sites that tested AL, day 3 parasitaemia was prevalent in only one site (SMH) at a rate of 2.3% (1/43), yielding a national rate of 0.2% (1/462). ETF was reported in three sites: one site after treatment with AS–AQ and two sites after treatment with AL (Table 3), yielding a national rate of 0.2% (1/492) for AS–AQ and 0.6% (3/472) for AL (p = 0.634) (Table 3). Nature of the ETF associated with AS–AQ treatment was parasitaemia on day 2 higher than day 0 whilst nature of the three ETF associated with AL treatment was parasitaemia on day 3 with axillary temperature > 37.5 °C (1 child) and day 2 parasitaemia higher than day 0 (2 children).

**Table 3:** Day 28 PCR-uncorrected study endpoints for children enrolled

Antimalarial drug	Treatment outcome	Sentinel site										Total
		WRH	NWMH	YMH	SMH	BMH	BGH	HMH	TAGH	LEKMH	EWP	
AS–AQ		n = 51	n = 55	n = 0	n = 54	n = 63	n = 62	n = 61	n = 61	n = 23	n = 62	N = 492
	ETF	0	1	–	0	0	0	0	0	0	0	1
	LCF	0	0	–	0	0	0	0	0	0	0	0
	LPF	0	0	–	0	1	1	0	0	0	4	6
	ACPR	45	54	–	51	62	58	58	60	20	58	466
	LFU	5	0	–	3	0	3	3	1	3	0	18
	WTH	1	0	–	0	0	0	0	0	0	0	1

Antimalarial drug	Treatment outcome	Sentinel site										Total
		WRH	NWMH	YMH	SMH	BMH	BGH	HMH	TAGH	LEKMH	EWP	
AL		n = 0	n = 58	n = 60	n = 43	n = 63	n = 63	n = 60	n = 58	n = 0	n = 67	N = 472
	ETF	–	0	0	1	0	0	0	2	–	0	3
	LCF	–	0	0	0	1	1	0	0	–	0	2
	LPF	–	5	3	3	3	1	0	12	–	9	36
	ACPR	–	53	54	39	59	57	47	42	–	54	405
	LFU	–	0	0	0	0	2	13	2	–	1	18
	WTH	–	0	3	0	0	2	0	0	–	3	8

*WRH* Wa Regional Hospital, *NWMH* Navrongo War Memorial Hospital, *YMH* Yendi Municipal Hospital, *SMH* Sunyani Municipal Hospital, *BMH* Bekwai Municipal Hospital, *BGH* Begoro Government Hospital, *HMH* Hohoe Municipal Hospital, *TAGH* Tarkwa Apinto Government Hospital, *LEKMH* Ledzokuku-Krowor Municipal Hospital, *EWP* Ewim Polyclinic, *ETF* early treatment failure, *LCF* late clinical failure, *LPF* late parasitological failure, *ACPR* adequate clinical and parasitological response, *LFU* loss to follow-up, *WTH* withdrawn

Per protocol analysis of treatment outcomes for 473 and 446 evaluable children who received AS–AQ and AL, respectively, showed day 28 PCR-uncorrected cure rates ranging between 93.5% (95% CI 84.3–98.2) and 100% for AS–AQ (national average of 98.5%; 95% CI 96.8–99.4) and between 75% (95% CI 61.6–85.6) and 100% for AL (national average of 90.8%; 95% CI 87.6–93.3). PCR-corrected cure rates ranged between 96.7% (95% CI 88.5–99.6) and 100% for AS–AQ and between 91.3% (95% CI 79.2–97.6) and 100% for AL (Fig. 1). The PCR-corrected national cure rates were 99.2% (95% CI 97.7–99.7) for AS–AQ and 96% (95% CI 93.5–97.6) for AL (p = 0.003). Site-specific cure rates for sites that studied both AS–AQ and AL showed no significant differences between the two drugs: NWMH (98.2% vs. 98.1%; p = 0.502); SMH (100% vs. 92.9%; p = 0.179); BMH (100% vs. 96.7%; p = 0.464); BGH (98.3% vs. 100%; p = 0.989); HMH (100% vs. 100%); TAGH (100% vs. 91.3%; p = 0.070); and EWP (96.7% vs. 91.5%; p = 0.415). Day 28 cumulative cure rates per Kaplan–Meier analysis showed similar rates as per protocol analysis (Table 4).

**Table 4:** Site-specific day 28 PCR-uncorrected and PCR-corrected cure rates (per protocol and Kaplan–Meier)

Site	PCR-uncorrected								PCR-corrected							
	AS–AQ				AL				AS–AQ				AL			
	PP		KM		PP		KM		PP		KM		PP		KM	
	n	% (95% CI)	n	% (95% CI)	n	% (95% CI)	n	% (95% CI)	n	% (95% CI)	n	% (95% CI)	n	% (95% CI)	n	% (95% CI)
NWMH	55	98.2 (90.3–100.0)	55	98.2 (87.8–99.7)	58	91.4 (81.0–97.1)	58	91.4 (80.5–96.3)	55	98.2 (90.3–100.0)	55	98.2 (87.8–99.7)	54	98.1 (90.1–100.0)	54	98.1 (87.6–99.7)
WRH	45	100.0 (92.1–)	51	100.0 (n/a)	–	–	–	–	45	100.0 (92.1–)	51	100.0 (n/a)	–	–	–	–
YMH	–	–	–	–	57	94.7 (85.4–98.9)	60	94.7 (84.6–98.3)	–	–	–	–	56	96.4 (87.7–99.6)	59	96.4 (86.5–99.1)
SMH	51	100.0 (93.0–)	54	100.0 (n/a)	43	90.7 (77.9–97.4)	43	90.7 (77.1–96.4)	51	100.0 (93.0–)	54	100.0 (n/a)	42	92.9 (80.5–98.5)	42	92.9 (79.5–97.6)
BMH	63	98.4 (91.5–100.0)	63	98.4 (89.3–99.8)	63	93.7 (84.5–98.2)	63	93.7 (84.0–97.6)	62	100.0 (94.2–)	63	100.0 (n/a)	61	96.7 (88.7–99.6)	61	96.7 (87.5–99.2)
BGH	59	98.3 (90.9–100.0)	62	98.3 (88.6–99.8)	59	96.6 (88.3–99.6)	63	96.6 (87.1–99.1)	59	98.3 (90.0–100.0)	62	98.3 (88.6–99.8)	57	100.0 (93.7–)	61	100.0 (n/a)
HMH	58	100.0 (93.8–)	61	100.0 (n/a)	47	100.0 (92.5–)	60	100.0 (n/a)	58	100.0 (93.8–)	61	100.0 (n/a)	47	100.0 (92.5–)	60	100.0 (n/a)
TAGH	60	100.0 (94.0–)	61	100.00 (n/a)	56	75.0 (61.6–85.6)	58	75.4 (62.0–84.6)	60	100.0 (94.0–)	61	100.0 (n/a)	46	91.3 (79.2–97.6)	53	92.2 (80.4–97.0)
LEKMH	20	100.0 (83.2–)	23	100.0 (n/a)	–	–	–	–	20	100.0 (83.2–)	23	100.0 (n/a)	–	–	–	–
EWP	62	93.5 (84.3–98.2)	62	93.5 (83.7–97.5)	63	85.7 (74.6–93.3)	67	85.7 (74.3–92.3)	60	96.7 (88.5–99.6)	62	96.8 (87.7–99.2)	59	91.5 (81.3–97.2)	65	91.7 (81.2–96.5)
Overall	473	98.5 (96.8–99.3)	492	98.5 (96.9–99.3)	446	90.8 (87.6–93.2)	472	90.9 (87.9–93.2)	470	99.2 (97.7–99.7)	492	99.2 (97.8–99.7)	422	96.0 (93.5–97.6)	455	96.1 (93.8–97.5)

*PP* per protocol, *KM* Kaplan–Meier, *WRH* Wa Regional Hospital, *NWMH* Navrongo War Memorial Hospital, *YMH* Yendi Municipal Hospital, *SMH* Sunyani Municipal Hospital, *BMH* Bekwai Municipal Hospital, *BGH* Begoro Government Hospital, *HMH* Hohoe Municipal Hospital, *TAGH* Tarkwa Apinto Government Hospital, *LEKMH* Ledzokuku-Krowor Municipal Hospital, *EWP* Ewim Polyclinic, *n/a* not applicable

### Secondary outcomes

**Fig. 2:**
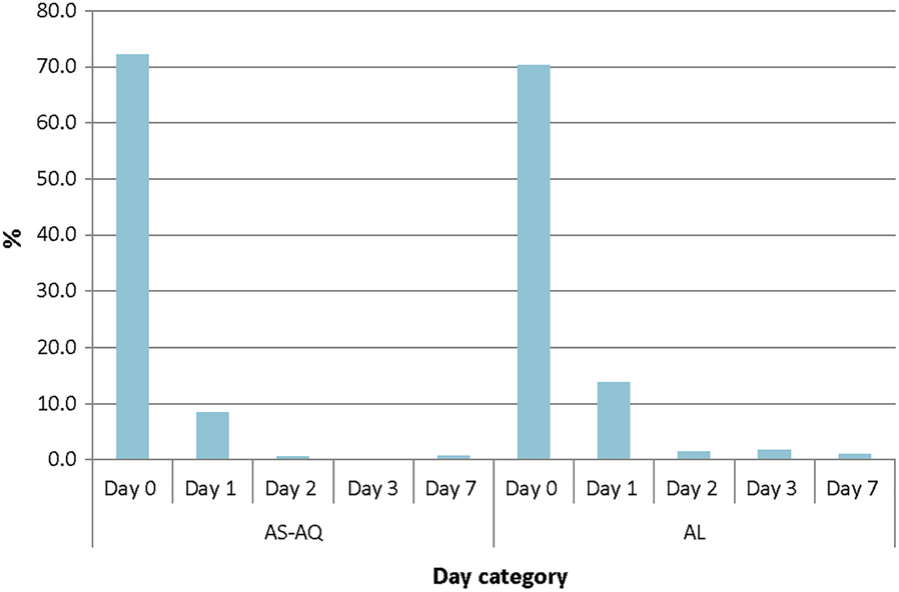
Proportion of children with measured fever (temperature ≥ 37.5 °C) during first week of follow-up

The overall proportion of children with axillary temperature ≥ 37.5 °C significantly declined after the first day of treatment with either AS–AQ (from 72.2%; 95% CI 68.0–76.1 to 8.4%; 95% CI 6.1–11.4; p < 0.001) or AL (from 70.3%; 95% CI 65.9–74.4 to 13.8%; 95% CI 10.9–17.4; p < 0.001) (Fig. 2). The significant decline was observed in all sites. However, the overall proportion of children with axillary temperature ≥ 37.5 °C after first day of treatment was significantly higher among those who received AL compared with those who received AS–AQ (13.8%; 95% CI 10.9–17.4 vs. 8.4%; 95% CI 6.1–11.4; p = 0.011). By day 7 there was no significant difference between AS–AQ and AL in terms of proportion of children with axillary temperature ≥ 37.5 °C (0.7% vs. 1.1%; p = 0.778) (Fig. 2).

**Fig. 3:**
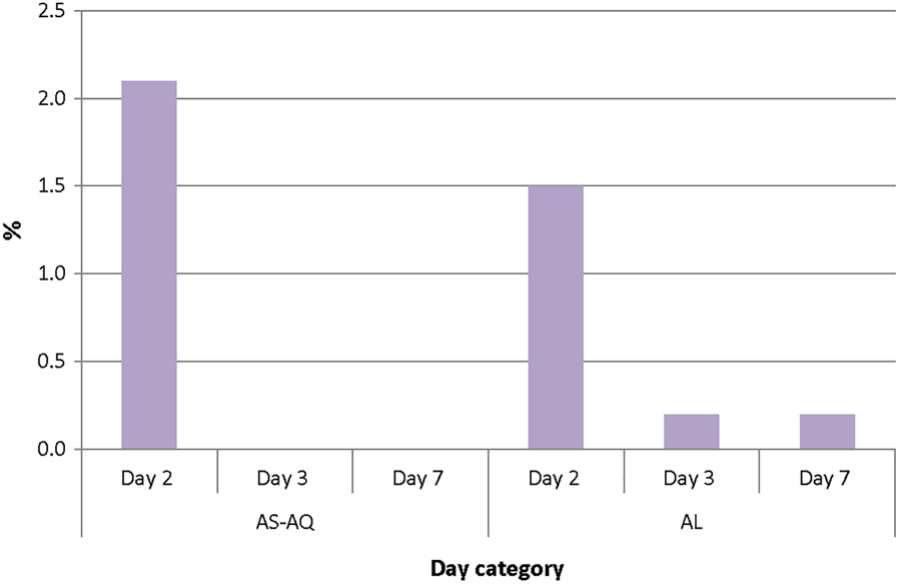
Proportion of children with parasites during first week of follow-up

**Fig. 4:**
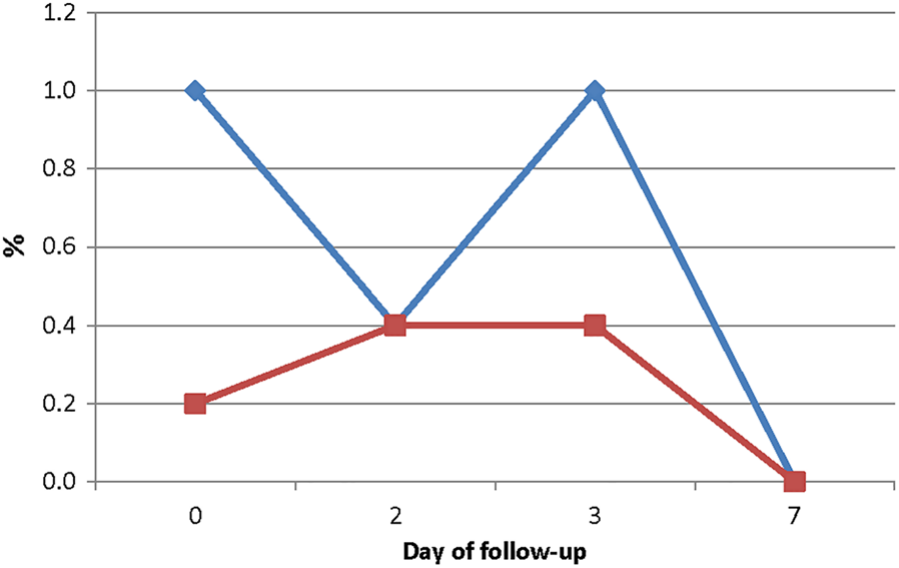
Proportion of children with gametocytaemia during first week of follow-up. Dark blue line represents AS–AQ and dark red line represents AL

The overall proportion of children with parasitaemia on day 2 following treatment with AS–AQ was not significantly different from children treated with AL (2.1% vs. 1.5%; p = 0.655) (Fig. 3). This pattern was observed in all sites that studied both AS–AQ and AL. Subsequently, the proportion of children with parasitaemia on day 3 and day 7 following treatment with AS–AQ was not significantly different from children treated with AL (0.0% vs. 0.2%; p = 0.956 for day 3 and 0.0% vs. 0.2%; p = 0.947 for day 7) (Fig. 3). Although pre-treatment (day 0) gametocytaemia was prevalent among children in the two treatment groups, there was no evidence of gametocytaemia in either group by day 7 post-treatment (Fig. 4).

The overall mean haemoglobin concentration significantly increased on day 28 compared with day 0 following treatment with either AS–AQ (10.1 g/dl ± 1.8 vs. 11.1 g/dl ± 1.5; p < 0.001) or AL (10.1 g/dl ± 1.6 vs. 10.9 g/dl ± 1.2; p < 0.001. There was only one site, NWMH, located in the savannah zone with no significant increase in haemoglobin concentration following treatment with AS–AQ whilst two sites (TAGH, located in the forest zone and EWP, located in the coastal zone) showed no significant increase following treatment with AL (Table 5).

**Table 5:** Changes in mean haemoglobin levels after treatment with AS–AQ and AL in sentinel sites in Ghana

Antimalarial drug	Site	Mean Hb (sd)		p-value
		Day 0	Day 28	
AS–AQ	WRH	8.8 (1.8)	9.6 (1.2)	0.015
	NWMH	9.6 (1.6)	9.9 (1.2)	0.268
	SMH	10.0 (1.7)	11.0 (1.0)	0.008
	BMH	10.3 (2.0)	11.1 (1.6)	0.015
	BGH	10.9 (1.9)	11.7 (1.1)	0.006
	HMH	10.1 (1.4)	11.5 (0.9)	< 0.001
	TAGH	10.5 (1.6)	11.2 (1.2)	0.015
	LEKMH	9.9 (1.7)	11.5 (0.8)	< 0.001
	EWP	10.8 (1.4)	12.1 (1.7)	< 0.001
	Overall	10.1 (1.8)	11.1 (1.5)	< 0.001
AL	NWMH	9.5 (1.6)	10.6 (1.2)	0.001
	YMH	9.0 (1.5)	10.4 (1.5)	< 0.001
	SMH	10.5 (1.2)	11.3 (0.8)	0.001
	BMH	9.7 (1.3)	10.8 (0.8)	< 0.001
	BGH	10.4 (1.4)	11.4 (1.1)	< 0.001
	HMH	10.2 (1.1)	10.9 (0.9)	0.001
	TAGH	10.9 (1.8)	11.4 (1.4)	0.103
	EWP	10.8 (1.7)	10.9 (1.1)	0.693
	Overall	10.1 (1.6)	10.9 (1.2)	< 0.001

*WRH* Wa Regional Hospital, *NWMH* Navrongo War Memorial Hospital, *YMH* Yendi Municipal Hospital, *SMH* Sunyani Municipal Hospital, *BMH* Bekwai Municipal Hospital, *BGH* Begoro Government Hospital, *HMH* Hohoe Municipal Hospital, *TAGH* Tarkwa Apinto Government Hospital, *LEKMH* Ledzokuku-Krowor Municipal Hospital, *EWP* Ewim Polyclinic

The main adverse event reported was vomiting. Prevalence of vomiting during the three days of treatment declined with day for both AS–AQ and AL from day 0 to day 2. The proportions were, however, significantly higher among children who received AS–AQ compared with those who received AL: 17.3% vs. 10.9% on day 0 (p = 0.001); 8.2% vs. 1.5% on day 1 (p < 0.001); and 6.5% vs. 0.4% on day 2 (p < 0.001). No serious adverse event was reported.

## Discussion

Routine surveillance on the therapeutic efficacy of anti-malarials in sentinel sites across Ghana has been ongoing since the introduction of ACT in 2005. For five rounds of surveillance conducted between 2005 and 2013, each of the 10 sites representing the 10 regions of the country were scheduled to study either AS–AQ or AL. For the purpose of obtaining site-specific efficacy data for both AS–AQ and AL in one round of surveillance, each sentinel site was scheduled to test both anti-malarials during the 2015–2017 round of surveillance. Seven of the 10 sites successfully tested both AS–AQ and AL whilst two sites tested only AS–AQ and another site tested only AL. The primary outcomes assessed were: (i) prevalence of day 3 parasitaemia, which is an indicator of suspected artemisinin (partial) resistance; and, (ii) a day 28 treatment outcome, which is an indicator of partner drug resistance [24]. Secondary outcomes assessed were patterns of fever and parasite clearance as well as changes in haemoglobin levels and prevalence of adverse events.

The study showed no presence of parasitaemia on day 3 following treatment with AS–AQ in all sites whilst only one person (0.2%) was parasitaemic on day 3 following treatment with AL. This finding is far below the WHO threshold of 10%, and therefore suggests that artemisinin (partial) resistance following ACT treatment of falciparum malaria is not a problem in Ghana [24].

The overall day 28 cure rates were 99.2% for AS–AQ and 96.0% for AL, yielding ACT treatment failure rates of between 0.8 and 4.0%. The ACT treatment failure rates observed are below the WHO threshold of 10%, and therefore suggest that amodiaquine and lumefantrine are not failing as partner drugs in ACT use in Ghana [24]. The high ACT cure rates compare well with other findings in previous studies in the country and other parts of Africa [9, 11–14, 25–32]. Although this study was not a comparative one, it was observed that in the seven sites that studied both AS–AQ and AL there were no statistically significant differences in cure rates even though the overall cure rate for AS–AQ was significantly higher than that of AL (99.2% vs. 96.0%; p = 0.003). This observation of seemingly significant difference between the overall cure rates of AS–AQ and AL when site-specific rates did not show such significant difference, raises the need to consider site-specific efficacy data in national anti-malarial drug policy reviews.

Both AS–AQ and AL showed rapid fever and parasite clearance during the first week of follow-up in all sites. Overall prevalence of fever declined by 88.4 and 80.4% after first day of treatment with AS–AQ and AL, respectively, whilst prevalence of parasitaemia on day 2 was 2.1% for AS–AQ and 1.5% for AL. Gametocytaemia was absent on day 7 following treatment with either AS–AQ or AL. These findings suggest that ACT remains effective in rapidly clearing fever and asexual parasites as well as reducing gametocyte carriage rates in Ghana [12–14, 25–32].

Generally, post-treatment mean haemoglobin concentration following treatment with either AS–AQ or AL was higher than pre-treatment concentration. Only one of the nine sites that studied AS–AQ and two of the eight sites that studied AL did not show a significant increase in post-treatment haemoglobin concentration. It is worth noting that all the three sites that did not show significant increase in post-treatment haemoglobin concentration following treatment with either AS–AQ or AL showed increases of between 0.1 and 0.5 g/dl, suggesting that ACT treatment has a positive impact on post-treatment haemoglobin levels as shown in previous studies [11, 25–32].

A commonly reported adverse event following treatment with either AS–AQ or AL was vomiting. Prevalence of vomiting on each day of treatment was significantly higher among children treated with AS–AQ compared with those treated with AL. This finding suggests that AL may be more tolerable than AS–AQ influencing patient preference and use as reported by Chatio and colleagues [33].

The main limitation of this study is the lack of pharmacokinetic data to better explain the recrudescence observed. The cure rates for AS–AQ and AL may therefore be higher than the rates observed. Furthermore, in view of the fact that this study was not a randomized trial, it cannot be concluded that AS–AQ is superior to AL even though its overall cure rate appeared to be higher than that of AL.

## Conclusions

The therapeutic efficacy of AS–AQ and AL are over 90% in sentinel sites across Ghana. The two anti-malarial drugs therefore remain efficacious in the treatment of uncomplicated malaria in the country achieving rapid fever and parasite clearance as well as low gametocyte carriage rates and improved post-treatment mean haemoglobin concentration.

## Additional file

**Additional file 1.** Participant flow showing number screened, enrolled and included in the per-protocol population.

## Data Availability

Data supporting the conclusions of this article are included within the article. The datasets analyzed are available upon reasonable request to the corresponding author.

## Abbreviations

ACT: artemisinin-based combination therapy
AS–AQ: artesunate–amodiaquine
AL: artemether–lumefantrine
DHAP: dihydroartemisinin–piperaquine
PCR: polymerase chain reaction
WHO: World Health Organization
NMWH: Navrongo War Memorial Hospital
WRH: Wa Regional Hospital
YMH: Yendi Municipal Hospital
SMH: Sunyani Municipal Hospital
BMH: Bekwai Municipal Hospital
BGH: Begoro Government Hospital
HMH: Hohoe Municipal Hospital
TAGH: Tarkwa Apinto Government Hospital
LEKMH: Ledzokuku-Krowor Municipal Hospital
EWP: Ewim Polyclinic
ETF: early treatment failure
LPF: late parasitological failure
LCF: late clinical failure
ACPR: adequate clinical and parasitological response
IRB: Institutional Review Board
GHS: Ghana Health Service
NMIMR: Noguchi Memorial Institute for Medical Research

## References

[1] Ghana Statistical Service. Ghana multiple indicator cluster survey with an enhanced malaria module and biomarker, 2011, final report. Accra: Ghana Statistical Service; 2011. http://www2.statsghana.gov.gh/docfiles/publications/MICS4_MAIN_REPORT.pdf.

[2] Ghana Statistical Service. Ghana malaria indicator survey, 2016. Accra: Ghana Statistical Service; 2017. http://www2.statsghana.gov.gh/docfiles/publications/MIS26.pdf.

[3] Ghana Health Service (2014). National Malaria Control Programme 2013 annual report.

[4] Ghana Health Service. National Malaria Control Programme 2017 annual report. Accra: Ghana Health Service; 2018. http://www.ccmghana.net/index.php/strategic-plans-reports?download=200:nmcp-2017.

[5] Ghana Health Service (2017). National Malaria Control Programme 2016 annual report.

[6] Nonvignon J, Aryeetey GC, Malm KL, Agyemang SA, Aubyn VNA, Peprah NY (2016). Economic burden of malaria on businesses in Ghana: a case for private sector investment in malaria control. Malar J..

[7] Ministry of Health, Ghana. Guidelines for case management of malaria in Ghana. 3rd ed. Accra: Ministry of Health; 2014. https://www.ghanahealthservice.org/downloads/GUIDELINEFORCASEMANAGEMENT.pdf.

[8] Nonvignon J, Aryeetey GC, Issah S, Ansah P, Malm KL, Ofosu W (2016). Cost-effectiveness of seasonal malaria chemoprevention in upper west region of Ghana. Malar J..

[9] WHO (2018). World malaria report 2018.

[10] WHO (2015). Guidelines for the treatment of malaria.

[11] Koram KA, Quaye L, Abuaku B (2008). Efficacy of amodiaquine–artesunate combination for uncomplicated malaria in children under five years in Ghana. Ghana Med J.

[12] Abuaku B, Duah N, Quaye L, Quashie N, Koram K (2012). Therapeutic efficacy of artemether–lumefantrine combination in the treatment of uncomplicated malaria among children under five years of age in three ecological zones in Ghana. Malar J..

[13] Abuaku B, Quaye L, Quashie N, Quashie N, Koram KA. Managing antimalarial drug resistance in Ghana: the importance of surveillance. In: Koram KA, Ahorlu CSK, Wilson MD, Yeboah Manu D, Bosompem KM, editors. Towards effective disease control in Ghana: research and policy implications, vol. 1. Accra: University of Ghana reader series; 2014. p. 7–18. https://books.google.com.gh/books?isbn=9988647506.

[14] Abuaku B, Duah N, Quaye L, Quashie N, Malm K, Bart-Plange C (2016). Therapeutic efficacy of artesunate–amodiaquine and artemether–lumefantrine combinations in the treatment of uncomplicated malaria in two ecological zones in Ghana. Malar J..

[15] WHO (2009). Methods for surveillance of antimalarial drug efficacy.

[16] Kasasa S, Asoala V, Gosoniu L, Anto F, Adjuik M, Tindana C (2013). Spatio-temporal malaria transmission patterns in Navrongo demographic surveillance site, northern Ghana. Malar J..

[17] Asare EO, Amekudzi LK (2017). Assessing climate driven malaria variability in Ghana using a regional scale dynamical model. Climate.

[18] Appawu M, Owusu-Agyei S, Dadzie S, Asoala V, Anto F, Koram K, Rogers W, Nkrumah F, Hoffman SL, Fryauff DJ (2004). Malaria transmission dynamics at a site in northern Ghana proposed for testing malaria vaccines. Trop Med Int Health..

[19] Abonuusum A, Owusu-Daako K, Tannich E, May J, Garms R, Kruppa T (2011). Malaria transmission in two rural communities in the forest zone of Ghana. Parasitol Res.

[20] Kweku M, Liu D, Adjuik M, Binka F, Seidu M, Greenwood B (2008). Seasonal intermittent preventive treatment for the prevention of anaemia and malaria in Ghanaian children: a randomized, placebo controlled trial. PLoS ONE.

[21] Mensah-Brown HE, Abugri J, Asante KP, Dwomoh D, Dosoo D, Atuguba F (2017). Assessing the impact of differences in malaria transmission intensity on clinical and haematological indices in children with malaria. Malar J.

[22] Wooden J, Kyes S, Sibley CH (1993). PCR and strain identification in *Plasmodium falciparum*. Parasitol Today..

[23] WHO. Methods and techniques for clinical trials on antimalarial drug efficacy: genotyping to identify parasite populations. Geneva: World Health Organization; 2007. http://www.who.int/iris/handle/10665/43824.

[24] WHO. Artemisinin resistance and artemisinin-based combination therapy efficacy: status report. Geneva: World Health Organization; 2018. https://apps.who.int/iris/bitstream/handle/10665/274362/WHO-CDS-GMP-2018.18-eng.pdf.

[25] Koram KA, Abuaku B, Duah N, Quashie N (2005). Comparative efficacy of antimalarial drugs including ACTs in the treatment of uncomplicated malaria among children under 5 years in Ghana. Acta Trop.

[26] Abuaku BK, Mensah BA, Ofori MF, Myers-Hansen J, Derkyi-Kwarteng AB, Essilfie F (2017). Efficacy of artesunate/amodiaquine in the treatment of uncomplicated malaria among children in Ghana. Am J Trop Med Hyg.

[27] Davlantes E, Dimbu PR, Ferreira CM, Joao MF, Pode D, Félix J (2018). Efficacy and safety of artemether–lumefantrine, artesunate–amodiaquine, and dihydroartemisinin–piperaquine for the treatment of uncomplicated *Plasmodium falciparum* malaria in three provinces in Angola, 2017. Malar J..

[28] Roth JM, Sawa P, Makio N, Omweri G, Osoti V, Okach S (2018). Pyronaridine–artesunate and artemether–lumefantrine for the treatment of uncomplicated *Plasmodium falciparum* malaria in Kenyan children: a randomized controlled non-inferiority trial. Malar J.

[29] Mandara CI, Kavishe RA, Gesase S, Mghamba J, Ngadaya E, Mmbuji P (2018). High efficacy of artemether–lumefantrine and dihydroartemisinin–piperaquine for the treatment of uncomplicated falciparum malaria in Muheza and Kigoma Districts, Tanzania. Malar J..

[30] Raobela O, Andriantsoanirina V, Rajaonera DG, Rakotomanga TA, Rabearimanana S, Ralinoro F (2018). Efficacy of artesunate–amodiaquine in the treatment of falciparum uncomplicated malaria in Madagascar. Malar J.

[31] Dama S, Niangaly H, Djimde M, Sagara I, Guindo CO, Zeguime A (2018). A randomized trial of dihydroartemisinin–piperaquine versus artemether–lumefantrine for treatment of uncomplicated *Plasmodium falciparum* malaria in Mali. Malar J..

[32] Mårtensson A, Strömberg J, Sisowath C, Msellem MI, Gil JP, Montgomery SM (2005). Efficacy of artesunate plus amodiaquine versus that of artemether–lumefantrine for the treatment of uncomplicated childhood *Plasmodium falciparum* malaria in Zanzibar, Tanzania. Clin Infect Dis..

[33] Chatio S, Aborigo R, Adongo PB, Anyorigiya T, Akweongo P, Oduro A (2015). Adherence and uptake of artemisinin-based combination treatment for uncomplicated malaria: a qualitative study in northern Ghana. PLoS ONE.

